# Impact of COVID-19 on Physical Fitness in Central Indian Athletes Aged 20-30 Years: A Cross-Sectional Pilot Study

**DOI:** 10.7759/cureus.46126

**Published:** 2023-09-28

**Authors:** Nitin B Dhokane, Aman L Lonare, Narhari P Pophali, Jyotsana Bharshankar, Piyush Kherde, Shrikant S Karwate, Anup Kumar D Dhanvijay

**Affiliations:** 1 Physiology, Government Medical College, Sindhudurg, Sindhudurg, IND; 2 Physiology, Government Medical College, Nagpur, Nagpur, IND; 3 Physiology, Shri Vasantrao Naik Government Medical College, Yavatmal, Yavatmal, IND; 4 Physiology, All India Institute of Medical Sciences, Deoghar, Deoghar, IND

**Keywords:** central indian, covid-19, physical fitness, young athletes, pilot study

## Abstract

Background

Physical fitness is of utmost importance to athletes as it ensures better performance in competitive sports. Athletes who contracted COVID-19 frequently experienced persistent symptoms for weeks or months afterward. Due to the direct effects of COVID-19 infection on pulmonary, cardiovascular, and neurological systems, combined with the negative effects of isolation and inactivity, it has been observed that physical fitness decreases in individuals. This study aimed to evaluate the physical fitness of young athletes in the age group of 20 to 30 years after mild-to-moderate COVID-19 infection and compare them with unaffected athletes of the same age group.

Methodology

A field-based, cross-sectional, comparative study was conducted from July 2022 to August 2022 in Nagpur, India. Physical fitness levels of 50 young athletes in the age group of 20-30 years who never got infected with COVID-19 were compared to 50 athletes with a recent history of mild-to-moderate COVID-19 infection using the Harvard step test, breath-holding test, and peak expiratory flow rate measurement. Participants were included based on COVID-19 diagnosis using standard procedures and confirmation of recovery through negative reverse transcriptase polymerase chain reaction tests.

Results

Overall physical fitness of athletes who suffered from mild-to-moderate COVID-19 infection was significantly less than those who were not infected. Compared to their non-COVID-19 counterparts, the COVID-19-recovered athletes showed reduced physical fitness index (p < 0.0001 for males and p = 0.0003 for females), reduced peak expiratory flow rate (p < 0.0001 for males and p < 0.0001 for females), and reduced breath-holding time (p < 0.0001 for males and p < 0.0001 for females).

Conclusions

COVID-19 had a significant impact on various components of physical fitness which may potentially affect the athletic performance and overall well-being of young athletes.

## Introduction

The COVID-19 pandemic has not only posed a threat to public health but has also disrupted various aspects of society, including sports and physical activities. Young athletes, who are essential contributors to the sporting world, have been affected by the virus and its associated consequences. There are now consistent reports of athletes reporting persistent and residual symptoms many weeks to months after COVID-19 infection [1].

The main site of COVID-19 infection is the lungs, as the virus gets into the body through the respiratory pathway. The infection starts spreading in one lung and progressively into another leading to impairment of the lungs to swap oxygen and carbon dioxide [2]. Physical exercise is considered key for its general benefits as well as a method to help airway clearance [3]. All body organs must function properly for good physical fitness; the effects of COVID-19 infection on the lungs may cause complications. As the body’s need for oxygen increases with higher levels of physical activity, a reduction in oxygen consumption may result in decreased physical strength, capability, and endurance. Good physical fitness is the foundation of sports; an athlete with good physical fitness not only improves the efficiency of learning sports abilities but also reduces the risk of injuries and mishaps [4].

The purpose of this pilot study was to investigate the effects of COVID-19 infection on the physical fitness of young athletes aged 20 to 30 years. By examining the various components of physical fitness, this study aimed to provide preliminary insights into the potential long-term impact of COVID-19 on the athletic performance and overall well-being of this population.

## Materials and methods

This pilot study employed a cross-sectional design and was conducted over a two-month period from July 2022 to September 2022. The research was conducted at two locations: the Department of Physiology, Government Medical College, Nagpur, India, and the Ishwar Deshmukh College of Physical Education Practice Ground, Hanuman Nagar, Nagpur, India. The study enrolled a total of 50 young athletes (31 males and 19 females), aged 20 to 30 years, who had previously recovered from mild-to-moderate COVID-19 infections, verified through standard diagnostic procedures. An additional 50 athletes (30 males and 20 females), who had never contracted COVID-19, were selected as the control group for comparative analysis. Participants in both categories (cases and controls) were recruited using a convenient sampling method. The criteria for recovery from COVID-19 included the cessation of self-isolation for seven days and the confirmation of negative laboratory results via reverse transcriptase polymerase chain reaction (RT-PCR) testing. Accordingly, physical fitness was tested after seven days of isolation and a concurrent negative COVID-19 report.

The study included athletes who met the following inclusion criteria: individuals willing to participate, those aged between 20 and 30 of any gender, those who tested positive for COVID-19 via RT-PCR (cases), those who were never COVID-19 positive on RT-PCR testing (controls), and individuals engaging in regular physical exercise for the past three to five years. Exclusion criteria comprised individuals who were unwilling to participate; those not consistent with physical exercise; individuals with any physical or psychological illnesses; a history of smoking, alcohol, or drug consumption; and those with chronic illnesses.

Sample size calculation

The sample size was calculated using Cochran’s formula as follows: n = Z^2^ p (100-p) / d2 = 4pq/d^2^. Using the formula, the sample size for our study was calculated as 32. We considered a precision level of 10% and a prevalence of 10% for COVID-19 infection among athletes. Accordingly, we recruited 50 cases and 50 controls in the study.

Measurements

A comprehensive assessment of physical fitness components was conducted on each participant in the morning after light breakfast. The physical fitness components are discussed below.

Harvard Step Test

In 1943, the Harvard step test was developed to assess the aerobic fitness and development of an athlete’s cardiovascular system [5]. The test requires a gym bench (51 cm for males, 41 cm for females) and a stopwatch. The metronome was programmed to run at 90 beats per minute. A 40 cm high hardwood bench was employed. The participant was shown the stepping cycle in time with the step frequency. To begin, the participant placed one foot on the bench, and then the other; the first foot was then lowered to the floor, followed by the other, i.e., up-up-down-down. The test was expected to last five minutes. The participant was regularly checked for signs of discomfort or suggestions that the test be terminated. At the end of five minutes, the participant was asked to stop the maneuver, and the radial pulse was counted at intervals of 1-1½, 2-2½, and 3-3½ minutes of completing the test to evaluate the Physical Fitness Index (PFI). The PFI was evaluated using the following formula [6]: PFI = (Duration of test in seconds × 100) / (2 × sum of pulse count of 1-1 ½, 2-2½, 3-3½ minutes).

Peak Expiratory Flow Rate Measurement

Peak expiratory flow rate (PEFR) is the volume of air forcefully expelled from the lungs in one quick exhalation. It is a reliable indicator of ventilation adequacy as well as flow obstruction [7]. The test requires Wright’s peak flow meter. The athlete is made to do a fast forceful expiration on the Wright’s peak flow meter, and the assessment is done according to the markings on the meter.

Breath-Holding Test

This test is used as a rough index of the cardiopulmonary reserve, measured by the length of time a person can hold his or her breath [8]. Athletes who are unable to hold their breath for more than 20 seconds have a possibility of respiratory dysfunction. Breath-holding is a novel method to identify patients with a high risk of respiratory failure in COVID-19 [9]. The test requires a stopwatch.

Statistical analysis

GraphPad Prism 7.0 was used for data analysis. The data were expressed as mean and standard deviation (mean ± SD) and percentage (n%) in Microsoft Excel 2010. Data were tested using the Shapiro-Wilk test and were found to be normally distributed, and the sample size was above 30 to consider the data as normal. Between groups, the PFI, PEFR, and breath-holding time (BHT) were compared using the unpaired t-test as our data were normal in distribution [10]. The statistical significance level was accepted at p-values <0.05.

Ethical considerations

The study was approved by the Institutional Ethics Committee of the Government Medical College, Nagpur (reference ID: 2022-01498/EC/Pharmac/GMC/Nagpur). The study objectives were explained to participants in their mother tongue and their questions were answered. Only participants who signed an informed consent form were enrolled. Participation in the study was strictly voluntary and participants were free to decline to answer any question or totally withdraw if they so wished at any time.

## Results

The study conducted on a group of 50 young athletes, comprising 31 males and 19 females who had previously recovered from COVID-19, yielded preliminary but significant findings. These findings suggest that COVID-19 infection has a substantial impact on the physical fitness of these athletes.

**Figure 1:**
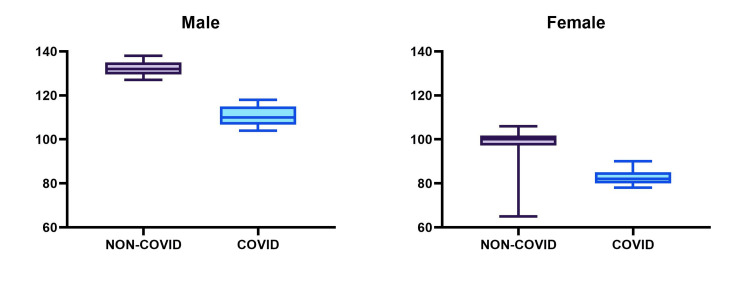
Physical fitness index according to gender and COVID-19 infection status.

**Figure 2:**
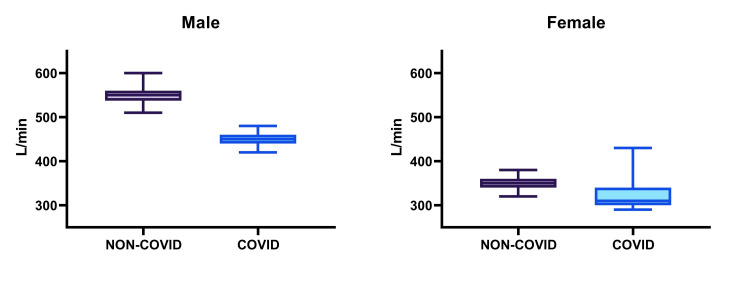
Peak expiratory flow rate according to gender and COVID-19 infection status.

**Figure 3:**
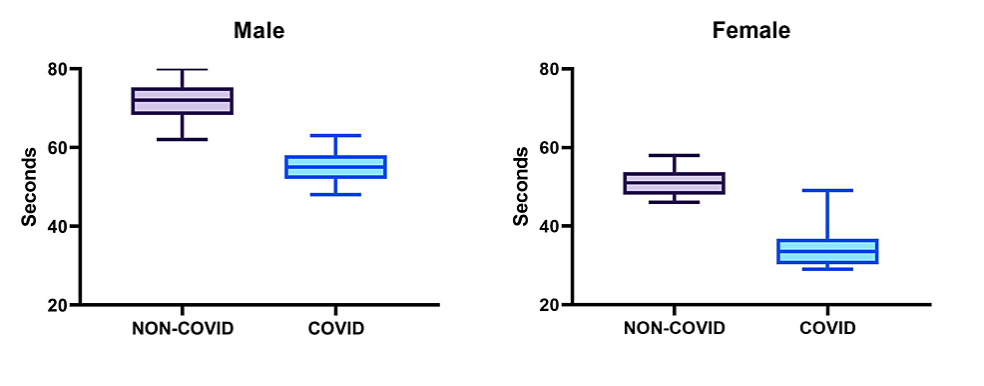
Breath-holding time according to gender and COVID-19 infection status.

The data, illustrated in Figures 1-3, reveal noticeable differences between the athletes who had contracted COVID-19 and those who had not. COVID-19-recovered athletes exhibited reduced aerobic capacity, as reflected in the PFI. For males, this index was significantly lower (132 ± 3.40 vs. 109.67 ± 5.53, p < 0.0001), and a similar pattern was observed among females (97.05 ± 11.22 vs. 78.63 ± 17.37, p = 0.0003). Regarding PEFR, the study revealed a decrease in ventilation adequacy, with COVID-19-recovered athletes displaying reduced PEFR values. In males, the difference was highly significant (547.67 ± 19.77 vs. 449.35 ± 13.34, p < 0.0001), and a comparable trend was observed in females (352 ± 16.73 vs. 316.31 ± 11.22, p < 0.0001). Regarding BHT, the cardiopulmonary reserve of COVID-19-recovered athletes was diminished, as evidenced by reduced BHT. In males, this reduction was pronounced (71.83 ± 5.14 vs. 54.96 ± 4.01, p < 0.0001), and a similar trend was observed among females (50.9 ± 3.35 vs. 33.73 ± 3.34, p < 0.0001). However, it is important to note that these results are preliminary, and further research is necessary to confirm and expand upon these findings.

## Discussion

Assessment of physical fitness does not always require heavy or expensive machinery, physical fitness can also be effectively assessed by some simple parameters and devices, such as those used in this study.

Harvard step test assesses the aerobic fitness and cardiovascular system of an individual. In males, a fitness level above 115 is considered excellent, and in females, a level above 91 is considered excellent [6]. In our study, athletes not infected by COVID-19 showed the highest PFI rating of excellent compared to non-COVID-19-infected athletes which fell in the moderate category of PFI. In males, the mean fitness index score significantly decreased by 17% in COVID-19-recovered athletes compared to non-infected athletes, whereas in females, the mean fitness index significantly decreased by 18.9% in COVID-19-recovered athletes compared to non-COVID-19 athletes. This shift is not extreme but still of concern, as mild-to-moderate COVID-19 infection may slightly lower the fitness index, but long and severe COVID-19 infection can severely reduce the PFI. Our results are in accordance with another study done by Śliż et al who studied the impact of COVID-19 infection on cardiorespiratory fitness in elite athletes and found that exercise performance deteriorated after COVID-19 [11]. Jafarnezhadgero and colleagues had similar findings when they studied the effect of recent COVID-19 infection in female recreational runners. COVID-19 has been implicated in lower running endurance and changed running kinetics, such as longer stance periods and weaker propulsive forces [12]. Due to its effects on the pulmonary, cardiovascular, and neurological systems, COVID-19 adversely affects the physical functions of individuals [13]. In the peripheral nervous system involvement, there are symptoms such as musculoskeletal pain, paraesthesia, ataxia, and muscle weakness. In central nervous system involvement, headache and vertigo are noted [14]. When all these symptoms that occur with COVID-19 infection are combined with the negative effects of isolation and inactivity, it has been observed that physical fitness decreases in individuals [15].

PEFR is a significant parameter for testing ventilation adequacy. Assessment of PEFR is important for the early detection and prevention of chronic obstructive pulmonary disease [16]. Males have higher PEFR scores than females because of higher lung capacity [17]. In our study, male athletes affected by COVID-19 infection had a 17.9% lower value of mean PEFR score compared to that of non-COVID-19-infected athletes, whereas in female athletes, the mean PEFR score of COVID-19-infected athletes was 10% lower than that of non-COVID-19-infected athletes. The decrease in PEFR scores in both sexes was statistically significant. This result of our study is similar to the study done by Bostancı and colleagues who in a prospective cohort study investigated the effect of COVID-19 infection on respiratory muscle strength and pulmonary function in unvaccinated athletes and reported similar results [18]. However, Komici et al. who investigated pulmonary function in post-COVID-19 competitive athletes reported different findings. Except for forced expiratory volume in the first second, pulmonary function was not impaired during the early recovery phase in a population of physically active adults. This may be because they had no comparison group in their study [19]. COVID-19 infection can cause lung function impairment, manifesting as restricted ventilation dysfunction, small airway dysfunction, and diffuse dysfunction, and therefore, decreases pulmonary functions as assessed by spirometry [20].

BHT involves holding of breath for the maximum possible time, and this duration is an indication of the cardiopulmonary reserve of an individual. An average human can hold their breath for 30 seconds, while athletes have the ability to hold their breath for more time, which indicates their huge cardiopulmonary reserve which plays a key role in performing physical activities [21]. Results from this study indicate that male athletes not infected by COVID-19 infection could hold breath for 23.4% more time than COVID-19-infected athletes. Similarly, non-COVID-19-infected female athletes could hold their breath for 33% more time than COVID-19-infected female athletes. The difference was statistically significant in both sexes. This finding in our study corresponds to another similar study done in India on non-athlete individuals who recovered from moderate COVID-19 infection [22]. BHT is influenced by several factors, including lung function, cardiovascular fitness, and overall health. COVID-19 can cause lung inflammation and damage, leading to decreased lung function in some individuals, especially those with severe or critical cases of the disease. This reduction in lung function may indirectly impact BHT [23]. Additionally, COVID-19 can cause systemic effects on the body, leading to fatigue and weakness, which might affect an individual’s ability to hold their breath for an extended period [24].

As can be seen, the negative effects of COVID-19 infection on the pulmonary, cardiovascular, and neurological systems and the restriction of physical activity due to the pandemic led to a decrease in some physical fitness parameters [25]. Preliminary findings of our study indicated that COVID-19 infection had a significant impact on the physical fitness of these athletes. Compared to the non-COVID-19 group, the COVID-19-recovered participants showed reduced cardiopulmonary efficiency (PFI), reduced ventilation adequacy (PEFR value), and reduced cardiopulmonary reserve (BHT). However, it is important to note that these results are preliminary, and further research is necessary to confirm and expand upon these findings.

## Conclusions

This pilot study provides initial insights into the effects of COVID-19 infection on the physical fitness of young athletes aged 20 to 30 years. The findings suggest that COVID-19 had a significant impact on various components of physical fitness, including cardiopulmonary efficiency, ventilation adequacy, and cardiopulmonary reserve. These observed changes may potentially affect the athletic performance and overall well-being of young athletes.

In conclusion, this pilot study highlights the need for further investigations into the impact of COVID-19 on physical fitness in young athletes. Understanding the long-term effects of the virus on athletes’ physical abilities and designing appropriate interventions will be crucial for their successful recovery and return to sports. By addressing these issues, we can better support the physical and mental well-being of young athletes in the post-pandemic era.
